# Combined effects of the bisphosphonate, zoledronic acid and the aromatase inhibitor letrozole on breast cancer cells *in vitro*: evidence of synergistic interaction

**DOI:** 10.1038/sj.bjc.6605579

**Published:** 2010-02-16

**Authors:** H L Neville-Webbe, R E Coleman, I Holen

**Affiliations:** 1 Academic Unit of Clinical Oncology, Cancer Research Centre, Weston Park Hospital, Sheffield S10 2SJ, UK

**Keywords:** zoledronic acid, letrozole, apoptosis, synergy, breast cancer

## Abstract

**Background:**

Aromatase inhibitors are widely used in the treatment of oestrogen receptor-positive post-menopausal breast cancer. These patients may also be receiving the bisphosphonate, zoledronic acid (ZA) to prevent bone loss or reduce skeletal morbidity in the setting of advanced disease. The potential biological interaction of these two drugs in breast cancer has not been assessed.

**Methods:**

Aromatase-expressing breast cancer cells were treated with letrozole and ZA either simultaneously or in sequence, and the resulting apoptosis was assessed by staining with Hoechst 33342 and propidium iodide and examined using a fluorescent inverted Leica DMIRB microscope and a UV filter.

**Results:**

We found that letrozole and ZA induce levels of apoptosis in breast cancer cells *in vitro* that are significantly greater compared with treatment with each drug alone. However, this potentially, synergistic relationship is drug-sequence dependent, occurring only when cells are treated with letrozole, followed by ZA. The converse sequence, or administering drugs simultaneously, induces levels of apoptosis no greater than each drug alone.

**Conclusion:**

Owing to the enhanced anti-tumour efficacy of sequential drug administration, our findings may indicate that, for post-menopausal women who require treatment with letrozole, ZA should also be considered.

Letrozole is a non-steroidal third-generation aromatase inhibitor (AI) that binds to aromatase, which controls the last step in oestrogen synthesis. The AIs induce a profound reduction in oestradiol, oestrone and oestrone sulphate levels and, as a result, inhibit the growth of oestrogen-dependent breast cancer. In post-menopausal breast cancer, AIs are increasingly used in both metastatic (Mouridsen *et al*, 2001) and adjuvant settings (BIG1-98 Collaborators 2009; Goss *et al*, 2005).

Breast cancer patients may also require bisphosphonate, zoledronic acid (ZA) for various reasons. For example, letrozole (as with other AIs) causes bone loss and increases the risk of osteoporosis (Lester and Coleman, 2005). In addition, there is growing evidence for an adjuvant function for bisposphonates in early breast cancer (Gnant *et al*, 2009). Finally, patients may also be receiving ZA for metastatic bone disease, to reduce skeletal morbidity and pain (Rosen *et al*, 2001). As a result, increasing numbers of patients are receiving both ZA and letrozole in combination, either in early or advanced breast cancer. Table 1 lists current trials involving both ZA and letrozole in breast cancer that are either actively recruiting patients or closed to recruitment, but with results awaited.

The ZA is a potent nitrogen-containing bisphosphonate that inhibits osteoclastic bone resorption (Green *et al*, 1994). All nitrogen-containing bisphosphonates inhibit farnesyl pyrophosphate synthase in the mevalonate pathway (Dunford *et al*, 2001), leading to inhibition of farnesyl pyrophosphate and geranylgeranyl pyrophosphate. These isoprenoids are required for the post-translation lipid modification (i.e. farnesylation and geranylgeranylation) of signalling GTPases, such as Ras, Rho and Rac (Rogers *et al*, 2000). As these control a variety of important osteoclast cell functions, their loss ultimately leads to osteoclast apoptosis through caspase-3 enzyme activation (Benford *et al*, 2001).

*In vitro*, ZA also affects cancer cells directly through a wide variety of mechanisms. These include induction of apoptosis, inhibition of cancer cell growth, reduction of cancer cell adhesion, invasion and anti-angiogenic effects. Many of these anti-tumour effects are specifically related to inhibition of the mevalonate pathway, with potential for even greater anti-tumour activity when combined with other drugs (reviewed by Winter *et al*, 2008). To date, the interactions between ZA and letrozole on breast cancer cells *in vitro* have not been studied.

We have investigated whether ZA and letrozole, using cell culture conditions that mimic the post-menopausal state, have the potential for synergistic induction of apoptosis in breast cancer cells *in vitro,* and present novel insights into the interaction of ZA and the AI, letrozole.

## Materials and methods

### Tissue culture

To study the effect of letrozole *in vitro*, the human breast cancer cell line, MCF7-Ca, was used (provided by Dr S Chen New York, USA). This is an oestrogen receptor- and aromatase gene-expressing breast cancer cell line. For experiments using ZA alone, cells were routinely maintained in RPMI 1640 media, supplemented with 10% foetal calf serum (FCS), glutamine (2 mM) (all purchased from Gibco, Invitrogen Corporation, Paisley, UK), penicillin (100 U ml^–1^), streptomycin (100 *μ*g ml^–1^) (both purchased from GlaxoSmithKline, Uxbridge, Middlesex, UK) and fungizone (Amphoteracin B) (5 *μ*g ml^–1^) (Invitrogen Life Technologies Ltd, Paisley, Renfrewshire, UK).

To mimic post-menopausal oestrogen levels, it was necessary to deplete the medium of available oestrogen and add the aromatase substrate, androstenedione (AD). Under these conditions the only oestrogen available to cells would be through conversion of AD to oestrogen by the aromatase enzyme. Therefore, cells were grown and treated in phenol-red-free, glutamine-free RPMI 1640 media (purchased from Gibco, Invitrogen Corporation), supplemented with dextran-coated charcoal-stripped (from Sigma-Aldrich, Poole, Dorset, UK) FCS. Phosphate-buffered solution (PBS) Dulbecco and trypsin-EDTA (0.05% trypsin, 0.53 mM EDTA.4Na in Hanks-buffered saline solution) were supplied by Gibco (Invitrogen Corporation). All plastics were purchased from Costar Ltd (Bucks, UK).

### Drugs and chemicals

The ZA ([1-hydroxy-2-(1H-imidazol-1-yl)ethylidene] bisphosphonic acid) was supplied as a disodium salt by Novartis (Basel, Switzerland). A stock solution (10 mM) was prepared in PBS and stored at −20°C. Letrozole was also a gift from Novartis and supplied as a dry powder that was reconstituted in 100% ethanol to make a 0.1 M stock solution, and stored at 4–8°C. The stock solution of each drug was diluted in appropriate culture media and sterile filtered before use.

Geranylgeraniol (GGOH) (all trans-3, 7,11-15-tetramethyl-2, 6,10,14-hexadecatetraen-1-ol) and 4-androsten-3, 17-dione (AD) were purchased from Sigma-Aldrich. Geranylgeraniol was diluted in ethanol (1 M stock solution) and stored at −20°C. For use in experiments, it was diluted at least 20 000-fold with culture media. Androstenedione was supplied as 1 mg dry powder, which was serially diluted in ethanol. A concentration of 25 nM was used for experiments on the basis of an earlier study by Professor A Brodie (Thiantanawat *et al*, 2003).

### Treatment of cells

Cells were seeded (at 20 000/0.5 ml per well) in 24-well plates and incubated for 3 days (to confluence) before treatment. On the day of treatment, cells were washed using 0.5 ml of sterile PBS, and an appropriate culture medium containing the drug(s) of interest was added to the wells.

### Proliferation assay

Cells (MCF7-Ca) were seeded in 24-well plates and treated with either letrozole (0–100 nM) or ZA (0–25 *μ*M), and at the end of each specified incubation period, cells were counted using a Coulter counter (Beckman Coulter, Z series).

### Apoptosis assay

At the end of the specified drug treatment period, cells were stained with 8 *μ*M Hoechst 33342 (Sigma RBI, Poole, Dorset, UK) and 5 *μ*M propidium iodide (Molecular Probes, Cambridge Biochemical, Cambridge, UK) for 15 min at 37°C. Both attached and detached cells were stained and examined using a fluorescent inverted Leica DMIRB microscope and a UV filter (355 nm excitation and 465 nm emission). Propidium iodide was excluded by viable cells, but stained the nuclei of necrotic cells. Hoechst 33342 entered the nuclei of viable and apoptotic cells, the latter being recognised by characteristic features of karyorrhexis. The incidence of viable, apoptotic and necrotic cells was obtained by counting using a Whipple graticule in five separate random grid areas in each of three wells for each treatment.

### Experiments involving sequential administration of ZA and letrozole

**Figure 1:**
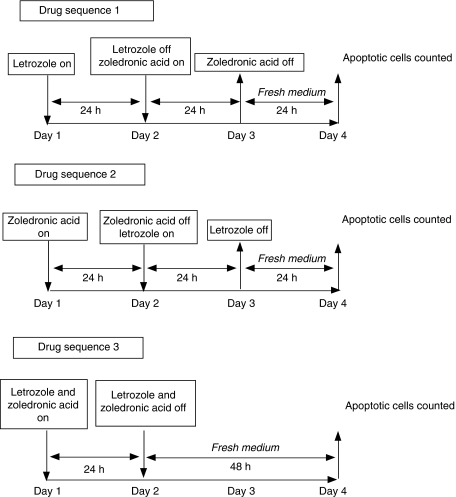
Experiment design for investigating the effects of sequential treatment of MCF7-Ca cells with letrozole (100 nM) and zoledronic acid (25 *μ*M). Drug sequence 1: ‘letrozole followed by zoledronic acid’ drug sequence 2: ‘zoledronic acid followed by letrozole’ drug sequence 3: ‘zoledronic acid and letrozole together’).

For these experiments, MCF7-Ca cells were seeded in 24-well plates, and cells were treated with ZA (10 *μ*M) and letrozole (100 nM), each for 24 h, alone and in combination, in oestrogen-free media supplemented with AD (25 nM), as follows (Figure 1 indicates the experiment design):

Group 1, ‘letrozole followed by ZA’: Cells were initially treated with letrozole on day 1, and after 24 h incubation (day 2), the cells were washed and then treated with ZA for 24 h until day 3. At the end of 24 h incubation with ZA, the cells were again washed and incubated in fresh media (FM) for a further 24 h, with cells being assessed for apoptosis and necrosis on day 4.

Group 2, ‘ZA followed by letrozole’: Cells were initially treated with ZA on day 1, and after 24 h incubation (day 2), they were washed and treated with letrozole for 24 h until day 3. At the end of 24 h incubation with letrozole, the cells were again washed and incubated in FM for a further 24 h, with cells being assessed for apoptosis and necrosis on day 4.

Group 3, ‘ZA and letrozole together’: Cells were treated with ZA and letrozole, both administered together on day 1. After 24 h (day 2), cells were washed and further incubated in FM until day 4.

Control groups: Cells were treated with negative and positive controls as follows:

with FM alone (days 1–4);

with ZA (days 1 and 2, added and removed at the same time as ZA in group 2 or 3, and 1, respectively, and cells further incubated in FM until day 4);

with letrozole (days 1 and 2, added and removed at the same time as letrozole in group 1 or 3, and 2, respectively, and cells further incubated in FM until day 4).

In all groups, cells were assessed for levels of viable, apoptotic and necrotic cells on day 4, as detailed in the above section.

### Statistical analysis

Statistical analysis was performed using SPSS 11 for Mac OS X. For analysis of differences between means, one-way analysis of variance or the Kruskal–Wallis test for non-parametric data was used. *Post hoc* analysis was carried out using Tukey's HSD, Dunnett's or the Games–Howell test, as appropriate. For non-parametric data, *post hoc* analysis was performed using the Mann–Whitney *U*-test, with a Bonferroni correction applied. All experiments were repeated at least three times, and graphs represent the mean of combined experiments with bars representing the s.e.m., unless otherwise indicated.

## Results

### Effect of letrozole on the growth and survival of MCF7-Ca breast cancer cells

**Figure 2:**
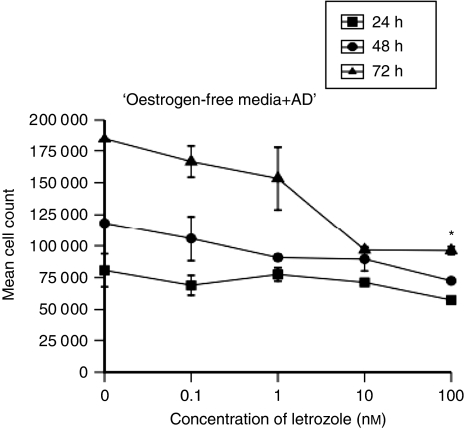
Growth inhibition of MCF7-Ca cells treated with letrozole (0–100 nM) for 24–72 h (^*^P=0.001 *vs* control).

**Figure 3:**
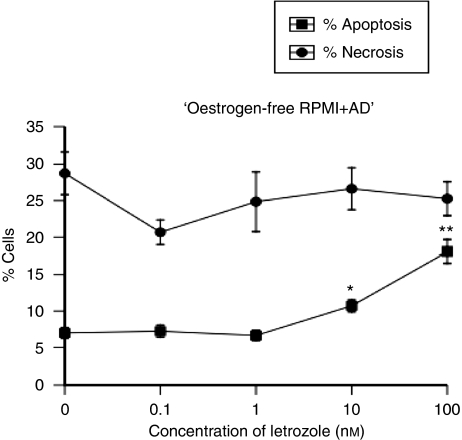
Induction of apoptosis *vs* necrosis in MCF7-Ca cells after treatment with letrozole (0–100 nM) for 72 h continuously in oestrogen-free media (^*^P<0.05 and ^**^P<0.001 *vs* control).

The MCF7-Ca cells were grown and treated with letrozole (0–100 nM) for up to 72 h in oestrogen-depleted media with addition of AD. Only after 72 h of treatment was there a significant growth inhibition with 100 nM (*P*=0.001 *vs* control), whereas 24 and 48 h of treatment with letrozole did not significantly inhibit cell growth (Figure 2). Treatment of cells with letrozole for 72 h, however, resulted in a significant dose-dependent increase in the percentage of apoptosis (*P*<0.0001) (Figure 2). A volume of 10 and 100 nM of letrozole induced 10.7 and 18.1% apoptosis compared with control levels of 7% (*P*=0.47 and *P*<0.001, respectively). It is interesting that, even with the highest concentration of letrozole, the levels of necrosis were not significantly greater than those in control samples, indicating that the mode of induced cell death of letrozole under these conditions is apoptosis (Figure 3).

### Effect of ZA on growth and survival of MCF7-Ca breast cancer cells

**Figure 4:**
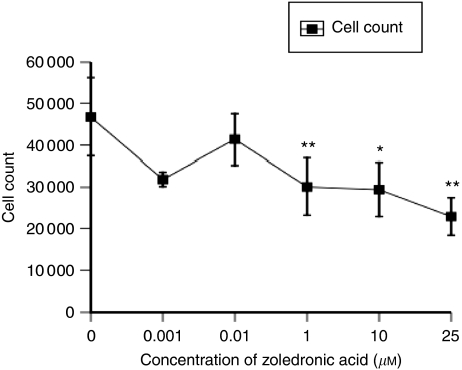
Growth inhibition of MCF7-Ca cells treated with zoledronic acid (0–25 *μ*M) for 1 h (^*^P<0.05 and ^**^P<0.01 compared with untreated controls).

In contrast to the growth-inhibitory effect of letrozole on MCF7-Ca cells, exposing the cells to ZA (0–25 *μ*M) for only 1 h induced significant growth inhibition with as little as 1 *μ*M of ZA. This caused a 35.8% reduction in cell proliferation, compared with that in control (*P*=0.0025). A similar growth-inhibitory effect was obtained with 10 *μ*M (37.2% reduction in proliferation, *P*=0.03) and 25 *μ*M (5.2% reduction in proliferation, *P*=0.005) (Figure 4).

**Figure 5:**
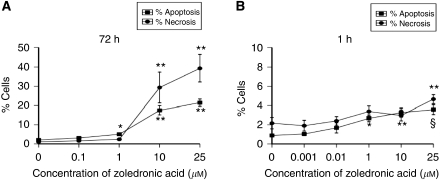
(**A**) Induction of apoptosis *vs* necrosis in MCF7-Ca cells after treatment with zoledronic acid (0–25 *μ*M) for 72 h continuously (^*^P<0.05 and ^**^P<0.01 *vs* control). (**B**) Induction of apoptosis *vs* necrosis in MCF7-Ca cells after treatment with zoledronic acid (0–25 *μ*M) for 1 h (^*^P<0.05, ^**^P⩽0.01 and ^§^*P*<0.001, compared with untreated controls).

To investigate the apoptosis-inducing effects of ZA, initial experiments investigated the effect of ZA (0–25 *μ*M) treatment of MCF7-Ca cells for 72 h continuously. This induced a significant concentration-dependent increase in apoptosis and necrosis, with a maximal level of 21.4% (*P*<0.01) and 39.3% (*P*<0.01), respectively, with 25 *μ*M (Figure 5A). Concentrations of at least 1 *μ*M of ZA induced a significant increase in apoptosis compared with control, whereas a concentration of at least 10 *μ*M was required to induce significant necrosis.

After a standard 4 mg infusion of ZA, serum concentrations rapidly fall within 1–2 h as the drug localises to the bone (Chen *et al*, 2002). When MCF7-Ca cells were treated with ZA (0–25 *μ*M) for 1 h, followed by a 72 h incubation in FM, there was still a concentration-dependent increase in apoptosis, compared with control. Apoptosis that was significantly greater compared with that in untreated controls occurred with at least 1 *μ*M. The maximal apoptosis induced was 3.6% with 25 *μ*M of ZA (*P*<0.001, compared with control). Significantly increased levels of necrosis were only caused by the highest concentration of ZA (25 *μ*M) (4.7 *vs* 2% in control, *P*=0.011) (Figure 5B).

Collectively, these results indicate that MCF7-Ca cells are sensitive to the apoptosis-inducing effects of ZA, even when used for a short period of 1 h. When extending the incubation period, there is a significant increase in the levels of apoptosis, but with an accompanying increase in necrotic cell death.

### Effect of sequential ZA and letrozole on induction of MCF7-Ca breast cancer cell apoptosis

We next investigated the combined effects of ZA and letrozole on MCF7-Ca cells, and whether drug sequencing had any effect on the levels of apoptosis. For these experiments, MCF7-Ca cells were treated with ZA (10 *μ*M) and letrozole (100 nM), each for 24 h, alone and in combination, in oestrogen-free media supplemented with AD (25 nM), as explained in the ‘Materials and Methods’ section.

**Figure 6:**
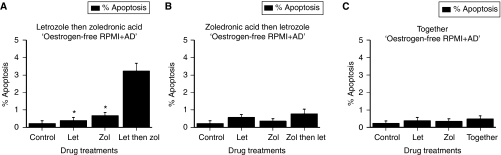
The effect of drug sequencing with ‘letrozole followed by ZA’ (**A**), ‘ZA followed by letrozole’ (**B**) and ‘ZA and letrozole together’ (**C**) on resultant apoptosis when MCF7-Ca cells are treated with letrozole (100 nM) and zoledronic acid (10 *μ*M), each for 24 h in oestrogen-free media plus AD (^*^P<0.001, compared with the drugs in combination).

As shown in Figure 6, increased levels of apoptosis occurred when cells were treated with letrozole and ZA. However, this was sequence dependent. Treating cells with letrozole, followed by ZA, induced a level of apoptosis that was eight times greater than treatment with letrozole alone (3.2 *vs* 0.4%, *P*<0.001) and nearly five times greater than that with ZA alone (3.2 *vs* 0.7%, *P*<0.001) (Figure 6A). However, treating cells with ZA, followed by letrozole, induced only a total of 0.8% apoptosis, which was not significantly greater than treatment with either drug alone (Figure 6B). Similarly, treating cells simultaneously with ZA and letrozole together induced only 0.4% apoptosis, again not significantly greater than that with either drug alone (Figure 6C).

Finally, the sequence ‘letrozole then ZA’ induced significantly more apoptosis than either the sequence ‘ZA then letrozole’ (*P*<0.001) or the sequence ‘ZA plus letrozole’ (*P*<0.001). In all three treatment groups (‘letrozole then ZA’, ‘ZA then letrozole’ and ‘ZA and letrozole together’), the levels of necrosis were not significantly different compared with those in untreated controls.

In summary, these results indicate that when MCF7-Ca cells are treated with letrozole and ZA in combination, only treating cells sequentially with letrozole followed by ZA induces substantially increased levels of apoptosis suggestive of synergy.

### Effects of low, clinically relevant concentrations of ZA

As discussed earlier, ZA leaves the circulation rapidly after intravenous infusion to localise to the bone, with plasma peak levels of around 1 *μ*M for 1 h (Chen *et al*, 2002). Consequently, having established that there is potential for enhanced levels of apoptosis when breast cancer cells are treated with letrozole followed by ZA, it was of interest to determine whether this effect could be achieved using a concentration of ZA that more accurately reflects peak circulating levels.

**Figure 7:**
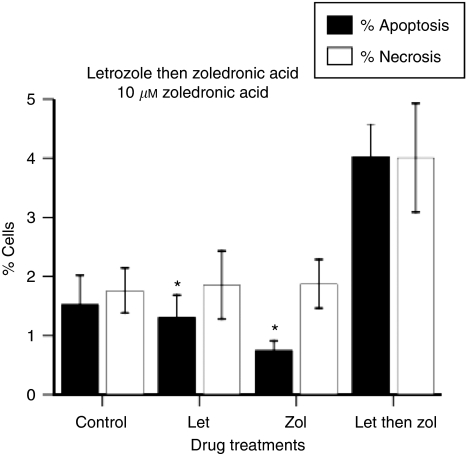
The effect of drug sequencing with letrozole followed by ZA on apoptosis when a clinically relevant concentration of ZA is used (^*^P<0.01 for letrozole followed by ZA *vs* each drug alone).

For initial experiments, MCF7-Ca cells were treated with letrozole (100 nM for 24 h), and thereafter with 10 *μ*M of ZA for 1 h only. We found that the resultant levels of apoptosis with sequential treatment was five times greater than that with ZA alone (4.0 *vs* 0.8%, *P*=0.001), and three times greater than treatment with letrozole alone (4.0 *vs* 1.3%, *P*=0.01) (Figure 7). However, when the concentration of ZA was further reduced to 1 *μ*M, treating MCF7-Ca cells with letrozole (100 nM for 24 h) followed by 1 *μ*M ZA for 1 h, the effect of enhanced apoptosis was not observed, with the resultant apoptosis being 1.3% and necrosis being 1.7%. In neither case was this significantly greater than treatment with each drug alone (data not shown). These results suggest a potentially synergistic relationship. Though not reproducible using a very low concentration of ZA, synergy is induced with 10 *μ*M, a concentration that could be achieved within the bone microenvironment.

### Function of the mevalonate pathway on ZA- and letrozole-induced apoptosis

To assess whether increased levels of apoptosis induced by ZA and letrozole in combination is, at least in part, due to inhibition of the mevalonate pathway by ZA, the effect of the addition of GGOH was investigated. Geranylgeraniol is a distal component of the mevalonate pathway. If the mechanism of induced apoptosis is through inhibition of this pathway, we would expect the levels of apoptosis to be restored when GGOH is added to cells being treated with ZA and letrozole. Cells (MCF7-Ca) were treated with letrozole (100 nM, 24 h), and thereafter with ZA (10 *μ*M, 1 h, with or without 50 *μ*M GGOH, added and removed simultaneously with ZA).

**Figure 8:**
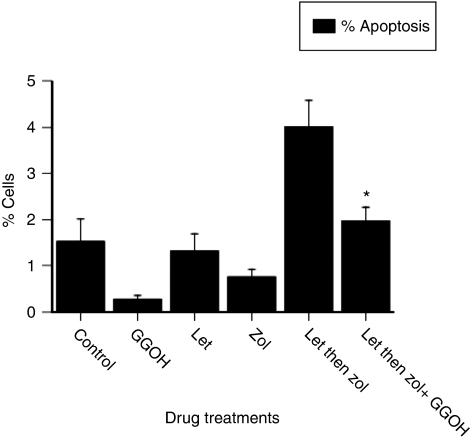
The effect of the mevalonate pathway intermediary, geranylgeraniol (GGOH) (50 *μ*M), on the apoptosis induced by treatment of MCF7-Ca cells with letrozole (100 nM, 24 h) followed by zoledronic acid (10 *μ*M, for 1 h) (^*^P=0.05 compared with ‘letrozole followed by ZA’).

For cells treated with letrozole followed by ZA, the resultant apoptosis was 4.0%, a level that was significantly greater than that caused by letrozole alone (1.3%, *P*=0.011), ZA alone (0.8%, *P*=0.001) or GGOH alone (0.3%, *P*<0.001) (Figure 8). The addition of GGOH to cells treated with letrozole followed by ZA reduced levels of apoptosis by 50% (2% with GGOH *vs* 4% without, *P*=0.05).

These results indicate that the increased level of apoptosis obtained when cells are treated with letrozole followed by ZA is, in part, through inhibition of the mevalonate pathway, as a component of this pathway, GGOH, restores levels of apoptosis by up to 50%.

## Discussion

We have evaluated the *in vitro* effects of different doses of letrozole and ZA, alone and in combination, on MCF7-Ca cell proliferation and apoptosis. As a single agent, letrozole alone significantly inhibited the growth of cells only after 72 h incubation with the highest tested concentration (100 nM). This suggests that growth inhibition by letrozole *in vitro* is not as important as the effects on apoptosis, as 100 nM of letrozole induced up to 18% apoptosis.

The ZA effectively inhibited the growth of these cells. Even just 1 h of treatment (followed by a drug-free incubation period) caused significant growth inhibition, with concentrations as low as 1 *μ*M. However, to detect this inhibitory effect, cells were incubated in fresh medium for up to 72 h after exposure to the drug. This period is required for a turnover of (already) prenylated proteins before inhibition of the mevalonate pathway by ZA (Neville-Webbe *et al*, 2005) and subsequent apoptosis.

In combination, ZA and letrozole have potential for synergistic induction of MCF7-Ca cells *in vitro*. However, increased induction of apoptosis only occurred when cells were treated with letrozole followed by ZA. ‘Letrozole then ZA’ induced a level of apoptosis that was over eight times greater than that caused by letrozole alone, and 4.8 times greater than treatment with ZA alone.

The ZA has consistently shown potential for synergistic interaction with drugs commonly used in breast cancer. This includes a combination with paclitaxel (Jagdev *et al*, 2001; Neville-Webbe *et al*, 2006), tamoxifen (Jagdev *et al*, 2000) and doxorubicin (Neville-Webbe *et al*, 2005). In our earlier study with ZA and chemotherapy drugs, we have similarly found that sequential administration of drugs is of paramount importance for inducing maximal (potentially synergistic) levels of apoptosis. For breast cancer cells treated with ZA in combination with either doxorubicin or paclitaxel, as with letrozole, cells had to be treated with the chemotherapy drug first, and thereafter with ZA. Treating cancer cells with the reverse sequence, or even simultaneously, was either antagonistic or, at best, additive, and the same patterns were seen here with letrozole. Our group recently evaluated this sequence-dependent synergy in an *in vivo* soft-tissue breast tumour model (Ottewell *et al*, 2008a, 2008b). Using clinically relevant doses of doxorubicin and ZA, inhibition of tumour growth was shown to be sequence dependent with doxorubicin followed by ZA, leading to inhibition of tumour growth associated with evidence of enhanced tumour cell apoptosis and reduced proliferation.

The biological effects of letrozole and ZA are largely mediated through the induction of apoptosis. It is likely that this is enhanced through inhibition of the mevalonate pathway. Using an intermediary of this pathway (GGOH), apoptosis was reduced by 50% for cells treated with the sequence ‘ letrozole then ZA’. Inhibition of the mevalonate pathway by nitrogen-bisphosphonates is integral for inducing a variety of effects including apoptosis of osteoclasts (Amin *et al*, 1992), breast cancer cells (Jagdev *et al*, 2001), prostate cancer cells (Oades *et al*, 2003), myeloma cells (Shipman *et al*, 1998) and inhibition of tumour cell invasion (Denoyelle *et al*, 2003). Furthermore, inhibition of this pathway also contributes to the apoptosis induced when breast (and prostate) cancer cells are treated in combination with ZA and doxorubicin (Neville-Webbe *et al*, 2005), or when breast cancer cells are treated with ZA and paclitaxel (Neville-Webbe *et al*, 2006).

Although *in vitro* and *in vivo* studies have limited implications for the clinical setting, it is interesting to note a recent update of the ZO-FAST study (Eidtmann *et al*, 2008). This clinical trial assesses the effects of ZA on letrozole-induced bone loss in post-menopausal women with early breast cancer. Patients are randomised to either combined letrozole and ZA (4 mg every 6 months) or letrozole alone. A recent update, with a 36-month follow-up, suggested a better outcome for those treated with the combination, with a 41% risk reduction in cancer recurrence. Although these results are preliminary, and the trial was not specifically set up to address the anti-tumour potential of ZA in patients receiving letrozole, they add to the growing evidence of the direct anti-cancer effects of this bisphosphonate in combination with other drugs, including endocrine treatments. Similarly, in the ABCSG 12 trial of pre-menopausal breast cancer patients (Gnant *et al*, 2009), 1803 patients received ovarian suppression with goserelin and were then randomised to receive either tamoxifen or the AI anastrazole, with or without ZA (4 mg infusions every 6 months for 3 years). No significant differences in disease-free survival were seen between tamoxifen and AI. However, the group receiving ZA had a statistically significant 36% reduction in the risk of disease recurrence, compared with patients receiving endocrine therapy alone (*P*=0.01). The reduction in metastatic events was not confined to a reduction in the frequency of bone metastases alone, but also applied to distant metastatic sites, loco-regional recurrence rates and cancer of contralateral breast. Reduction of metastases outside the skeleton adds to the growing evidence that the anti-tumour effects of ZA are not confined to the bone, and such anti-tumour effects may be enhanced by combination with other drugs.

In summary, letrozole has anti-tumour activity (which is largely through induction of apoptosis) when used alone under culture-media conditions that mimic the post-menopausal state. When combined with ZA, there is potential for a synergistic induction of apoptosis, but only when cancer cells are treated with letrozole followed by ZA. This may have positive implications for post-menopausal patients receiving both these drugs in the clinical setting.

## Figures and Tables

**Table 1:** Current trials involving zoledronic acid in conjunction with letrozole, actively recruiting or results awaited, in breast cancer patients

**Trial identification**	**Trial name**	**Phase**	**Sponsor**	**Number of patients**	**Status**
NCT00050011	Zoledronic acid – letrozole adjuvant synergy trial (ZFAST) – cancer treatment-related bone loss in post-menopausal women with oestrogen receptor positive and/or progesterone receptor positive breast cancer receiving adjuvant hormonal therapy	III	Novartis	602	Active, not recruiting, no results available
NCT00171314	The use of zoledronic acid to prevent cancer-treatment bone loss in post-menopausal women receiving adjuvant letrozole for breast cancer	III	Novartis	500	Completed, results not available as yet
NCT00171340	The use of zoledronic acid to prevent cancer-treatment bone loss in post-menopausal women receiving adjuvant letrozole for breast cancer	III	Novartis	900	Completed, results not available as yet
NCT00375752	Efficacy and safety of letrozole *vs* letrozole plus zoledronic acid as endocrine therapy before surgery in post-menopausal patients with breast cancer	II/III	Novartis	860	Currently recruiting
NCT00332709	Safety/efficacy of letrozole monotherapy or in combination with zoledronic acid as extended adjuvant treatment of post-menopausal patients with primary breast cancer	III	Novartis	460	Currently recruiting
NCT00247650	Randomized multi-centre study comparing prolonged primary systemic endocrine therapy with letrozole alone or in combination with zoledronic acid in early breast cancer (neoadjuvant study in Canada)	II	Novartis	190	Completed, no results available
NCT00376740	Phase 3 study of the effect of zoledronic acid in the prevention of osteoporosis in early breast cancer patients receiving the aromatase inhibitor, letrozole, in the adjuvant setting	III	Soroka University Medical Center	80	Currently recruiting
NCT00114270	Evaluating the effect of letrozole with or without concomitant zoledronic acid on oestrogen responsive targets in post-menopausal women	III	University of Virginia	120	Active, not recruiting
NCT00107263	A randomized, controlled, open-label trial of empiric prophylactic *vs* delayed use of zoledronic acid for prevention of bone loss in post-menopausal women with breast cancer initiating therapy with letrozole after tamoxifen	III	North Central Cancer Treatment Group	550	Active, not recruiting
NCT00412022	Phase III randomized study of the effects on bone mineral density of tamoxifen, letrozole and letrozole + zoledronic acid as adjuvant treatment of patients with early breast cancer	III	National Cancer Institute, Naples	500	Currently recruiting
NCT00436917	Zoledronic acid for treatment of osteopenia and osteoporosis in women with primary breast cancer undergoing adjuvant aromatase inhibitor (letrozole) therapy	Open-label	Mayo Clinic	60	Active, not recruiting
NCT00903162	Extended endocrine therapy for pre-menopausal women with breast cancer	Open-label	Dana-Farber Cancer Institute	50	Currently recruiting
NCT00072020	Does adjuvant zoledronic acid reduce recurrence in patients with high-risk localised breast cancer? (AZURE)^a^	III	University of Sheffield	3352	Awaiting results

a Patients may be also receiving chemotherapy or other endocrine agents, not exclusively letrozole, with or without zoledronic acid.

*Sources*: UK clinical trials gateway (http://www.controlled-trials.com) and Clinicaltrials.gov (http://www.clinicaltrials.gov.ct2/home).
